# Factors affecting final functional outcomes in open‐globe injuries and use of ocular trauma score as a predictive tool in Nepalese population

**DOI:** 10.1186/s12886-021-01819-4

**Published:** 2021-02-04

**Authors:** Saurav M. Shrestha, Casey L. Anthony, Grant A. Justin, Madhu Thapa, Jyoti B. Shrestha, Anadi Khatri, Annette K Hoskin, Rupesh Agrawal

**Affiliations:** 1 B.P. Koirala Lions Centre for Ophthalmic Studies, Institute of Medicine, Tribhuvan University Teaching Hospital, Kathmandu, Nepal; 2 Department of Vitreoretina, Mechi Eye Hospital, Birtamode, Nepal; 3 School of Medicine, Emory University, Atlanta, GA USA; 4 Department of Ophthalmology, Brooke Army Medical Center, San Antonio, TX USA; 5 Department of Surgery, Uniformed Services University of the Health Science, Bethesda, MD USA; 6 Department of Vitreoretinal services, Birat Eye Hospital, Biratnagar, Nepal; 7 Department of Ophthalmology, Birat Medical College and Teaching Hospital, Biratngar, Nepal; 8 Save Sight Institute, University of Sydney, Sydney, Australia; 9 Lions Eye Institute, University of Western Australia, Perth, Australia; 10 National Healthcare Group Eye Institute, Tan Tock Seng Hospital, Singapore, Singapore; 11 Singapore Eye Research Institute, Singapore, Singapore; 12 Moorfields Eye Hospital, NHS Foundation Trust, London, UK

**Keywords:** BETTS, Ocular trauma, Ocular trauma score, OGI, Open Globe Injury, OTS

## Abstract

**Background:**

Open globe injury (OGI) is one of the most devastating form of ocular trauma. The aim of the study is to identify the epidemiology and predict visual outcomes in traumatic open globe injuries using ocular trauma score (OTS) and correlate with final visual acuity (VA) at 3 months.

**Methods:**

Patients older than 5 years, presenting to B.P. Koirala Lions Centre for Ophthalmic Studies (BPKLCOS) from March 2016- March 2017 with OGI that met inclusion criteria were evaluated. Patient profile, nature and cause of injury, and time to presentation were recorded. Patients were managed accordingly and followed up to 3 months. An OTS score for each patient was calculated and raw scores were categorized accordingly. The VA after 3 months were compared to the predicted OTS values.

**Results:**

Seventy-three eyes of 72 patients were examined. 76 % were male, and the mean age was 26.17 years (median, 23.5 years). The mean time from injury to presentation was < 6 hours (30 patients, 41 %). Thirty-seven eyes (51 %) had zone I trauma, followed by twenty eyes (27 %) with zone II, and sixteen eyes (22 %) with zone III trauma. Sixty-five patients (90 %) were managed surgically, and fifty (68 %) received intravitreal antibiotics with steroid. When compared, the projected VA as per OTS were able to predict actual final visual outcomes in 60 % of the eyes with OGI of various zones.

**Conclusions:**

OTS can be an accurate predictive tool for final visual acuity even with a short follow up period of 3 months; with poor presenting visual acuity, delayed presentation, posterior zones of injury, need for intravitreal injections, endophthalmitis, and globe rupture associated with poorer prognosis.

## Background

Ocular trauma is one of the leading causes of ocular morbidity and monocular blindness in the world, with open globe injury (OGI) constituting a major portion of trauma related vision loss [1]. A loss of one eye equates to 24 % of whole-body disability, increasing to 85 % if the patient is bilaterally blind [2]. Hence, a major ocular injury can result in both severe physical damage and psychological trauma for patients and relatives. Approximately 750,000 cases of ocular trauma are hospitalized each year, with 203,000 open globe injuries per year worldwide [3, 4]. In Nepal, ocular trauma is the second leading cause of unilateral blindness after cataract, with 8.6/1,000 people having a history of ocular trauma [5]. Several studies have shown an increasing prevalence of ocular trauma with a bimodal distribution [6]. Males are six times more likely to be affected than females, and a recent report showed a shift from workplace to home as the place of injury [3, 7–11].

The prognosis of ocular injury cases, while varied, has improved in recent years due to the development of microsurgical and vitreoretinal techniques [10]; however, a historical lack of standard protocols and terminology made it difficult to appropriately triage and manage patients. The management of open globe injury is driven by a desire to achieve the best possible long-term visual outcome, and having prognostic information is important for triaging decisions and counseling a patient and their family. Functional prognosis following ocular injury varies widely with various risk factors associated with poorer visual outcomes [12–15].

The Birmingham Eye Trauma Terminology System (BETTS) is a widely accepted standardized system of categorizing ocular trauma terminologies that enables the accurate transmission of clinical information and study data [16, 17]. The Ocular Trauma Score (OTS) is a simplified predictive tool for ocular trauma cases first described by Kuhn et al. in 2002 [4, 18]. It is based on BETTS and the features of globe injury at initial examination, with scores based on risk factors shown to be associated with visual outcomes. The score’s predictive value is useful not only for counseling patients and families, but for managing expectations and guiding clinical decisions, particularly in resource-limited settings. Rationale for OTS being a reliable predictive tool would be valuable as the scoring system is classically used to predict the visual outcome of patients after ‘open-globe ocular trauma’ which by definition is a full thickness wound of the eye wall with the condition usually resulting to blindness [19]. Various studies have validated the OTS as a reliable predictive tool, with a predictive accuracy of up to 80 % [20, 21].

Multiple studies have reported a wide range of incidence of open globe injuries in diverse geographical settings, however only a few studies have addressed trauma including rural areas of developing country, and no studies have evaluated the extent of open globe injury  with the use of OTS in this type of setting in Nepal [7]. This hospital-based prospective study, which was conducted at one of the tertiary eye care centers in Nepal, aims to characterize OGI in the region, identify risk factors, correlate with visual outcomes, and implement and test the predictive value of OTS as a standard tool for management of OGI.

## Methods

A hospital-based prospective study was conducted at BPKLCOS under Tribhuvan University Teaching Hospital (TUTH) from March 2016- March 2017. This study received Institutional Review Board approval (Institute of Medicine, TUTH), and informed consent was obtained from each patient/ guardian and complied with the Tenets of the Declaration of Helsinki.

### Sample size and sampling technique

Non- probability purposive sampling technique was used. All diagnosed cases of OGI, irrespective of gender, laterality, duration of presentation, and other chronic systemic illness, that presented to BPKLCOS and the TUTH emergency department between March 2016 – March 2017 were included in the study. Patients with life-threatening conditions requiring life support, patients under 5 years of age, and those unwilling or unable to undergo ocular evaluations and investigations were excluded.

### Data collection

Detailed history and clinical examination were conducted for all patients. History from the patient or guardian included: chief complaint, mode and agent of trauma, place of injury, time to presentation, treatment prior to hospital presentation, and past medical and surgical history. The eye examination included assessment of visual acuity using Snellen visual acuity chart, direct and consensual pupillary reaction/ Relative afferent pupillary defect (RAPD), periocular, anterior segment, and posterior segment examination. Extent and type of ocular injury was defined complying the BETTS terminologies and classifications [17]. Zones of injury were classified based on Ocular Trauma Classification Group, where Zone I injuries involves only the cornea, Zone II injuries extends from limbus to anterior 5 mm of sclera and Zone III injuries extends beyond 5 mm from limbus in sclera [22]. All patients received an orbital x-ray and A/B scan, as well as CT orbit/head when indicated.

Predicted visual outcome was calculated for each patient using Ocular Trauma Score [4, 18]. On initial examination, a raw score depending on the initial visual acuity was assigned. The final score was calculated by subtracting points, according to presence or absence of various predefined variables, from the initial raw score. OTS variables includes Globe rupture, Endophthalmitis, Perforating injury, Retinal detachment (RD), and RAPD. The final score was matched to the relevant OTS group, ranging from 1 (most severe injury) to 5 (least severe injury) and are associated with a published range of predicted post-injury visual acuities, which correlates with an estimated probability of final visual acuity.

All patients were admitted to the eye ward of TUTH and managed surgically under general anesthesia as indicated. Intravitreal injections of Ceftazidime (2.25 mg/0.1 ml), Vancomycin (1 mg/0.1 ml) and Dexamethasone (0.4 mg/0.1 ml) were administered in eyes with posterior segment involvement – either in terms of extension of the injury, inflammation or radiological findings from the B-scan. Patients were clinically evaluated a minimum of once daily for one week and followed up weekly for one month and monthly for three months. At the end of 3 months follow up, the final visual acuity was compared with the OTS predicted visual acuity.

### Statistical analysis

Data entry, processing and statistical analysis of result was completed using SPSS software Version 20.0. Fisher’s exact test was used to compare categorical distribution of visual acuity in patient with or without various risk factors. Z-test for proportion was used for comparison of categorical distribution of final visual acuity with the OTS predicted visual acuity. McNemar’s Chi-square test was used to compare categorical distribution of final visual acuity with the presenting visual acuity. P values less than 0.05 were considered statistically significant.

## Results

### Epidemiology

Between March 2016- March 2017, a total of 95 patients presented with OGI. Twenty-three patients meeting various exclusion criteria were excluded from the study. Seventy-three eyes of 72 patients meeting inclusion criteria were included in the study cohort. Majority of the patients (55, 76 %) were male and mean age was 26.17 ± 19.12 years (min: 5, max: 80, median 23.50). Distribution according to occupation and geographic location is given in Table 1.

**Table 1:** Demographic profile of patients included in study

Parameters	Frequency (%)	Male	Female
**Age (years)**			
5–10	19 (26)	11	8
11–20	13 (18)	10	3
21–30	14 (19)	12	2
31–40	10 (14)	9	1
41–50	10 (14)	8	2
51–60	2 (3)	2	0
61–70	2 (3)	1	1
71–80	2 (3)	2	0
**Occupation**			
Student	28 (38)	20	8
Farmer	8 (11)	8	0
Carpenter	5 (7)	5	0
Service	5 (7)	5	0
Unemployed	7 (10)	3	5
Metal worker	4 (6)	4	0
Other	14 (18)	10	1
**Geographic distribution**			
Hilly region	60 (83)	46	14
Terai (lowland) regions	12 (17)	9	3

Largest number of patients belonged to age group of 5–10 years. Most of them were students and belonged to Hilly region of the country

### Pattern of ocular trauma

The majority of patients (58, 81 %) presented to the emergency department with a complaint of diminished vision. Right eye (40, 55 %) was affected more frequently than the left eye. Most injuries were caused by accidents at home (29, 40 %), with vegetative matters (17, 23 %) being the most common causative agent. Thirty-seven eyes (51 %) had injuries involving Zone I, while 20 eyes (27 %) and 16 eyes (22 %) had Zone II and Zone III injuries respectively [Table 2]. Thirty patients (41 %) presented within 6 hours of sustaining injury, while time to presentation was 6–24 hours in 23 patients (32 %), 1 day to 1 week in 15 patients (21 %), and > 1 week in 4 patients (6 %). A total of 65 patients (90 %) were managed surgically, with 20 patients (27 %) within 6 hours of presentation, 38 patients (53 %) in 6–24 hours, and 7 patients (10 %) at more than 24 hours. Of the 72 patients, 50 patients (68 %) received an intravitreal injection of Ceftazidime, Vancomycin and Dexamethasone, while 7 patients (10 %) received no surgical management.

**Table 2:** Patterns of ocular trauma

Parameters	Frequency (%)	Male	Female
**Affected eye**			
Right	40 (56)	31	9
Left	31 (43)	23	8
Bilateral	1 (1)	1	0
**Zones of involvement**			
Zone I	37 (51)	29	8
Zone II	20 (27)	12	8
Zone III	16 (22)	14 (1)^a^	1
**Mode of presentation**			
Emergency	58 (81)	44	14
OPD	14 (19)	11	3
**Place of trauma**			
Home	29 (40)	21	8
Field	13 (18)	11	2
Road	10 (14)	10	0
School	10 (14)	6	4
Workshop	5 (7)	4	1
Jungle/Garden	4 (6)	2	2
Shop	1 (1)	1	0
**Agent of trauma**			
Plant (vegetative material)	17 (23)	7 (1)^a^	9
Metal/Nails	15 (21)	14	1
Sharp object	12 (16)	10	2
Projectile	11 (15)	8	3
Furniture/Appliances	8 (11)	7	1
Blunt object	7 (10)	7	0
Body part	3 (4)	2	1
**Chief complaint**			
Diminution of vision	45 (61)	35	10
Ocular pain	21 (29)	17	4
Swelling of eye	4 (7)	2	2
Inability to open eye	2 (3)	1	1
**Mode of trauma**			
Accident	58 (80)	45	13
Physical assault	3 (4)	2	1
Inadvertent	6 (9)	3	3
RTA	5 (7)	5	0

*OPD* Outpatient department, *RTA* Road traffic accident

^a^Bilateral eye involvement of a single person

Among the 73 eyes, 23 (32 %) had hyphema in the anterior chamber and 36 (49 %) had lens injury. A total of 10 eyes (14 %) had a retained intraocular foreign body. Out of 73 eyes, 9 (12 %) had a ruptured globe, 9 (12 %) presented with or developed endophthalmitis, 7 cases (10 %) exhibited retinal detachment, and there was one case of a perforated globe.

### Ocular examination and OTS

**Fig. 1:**
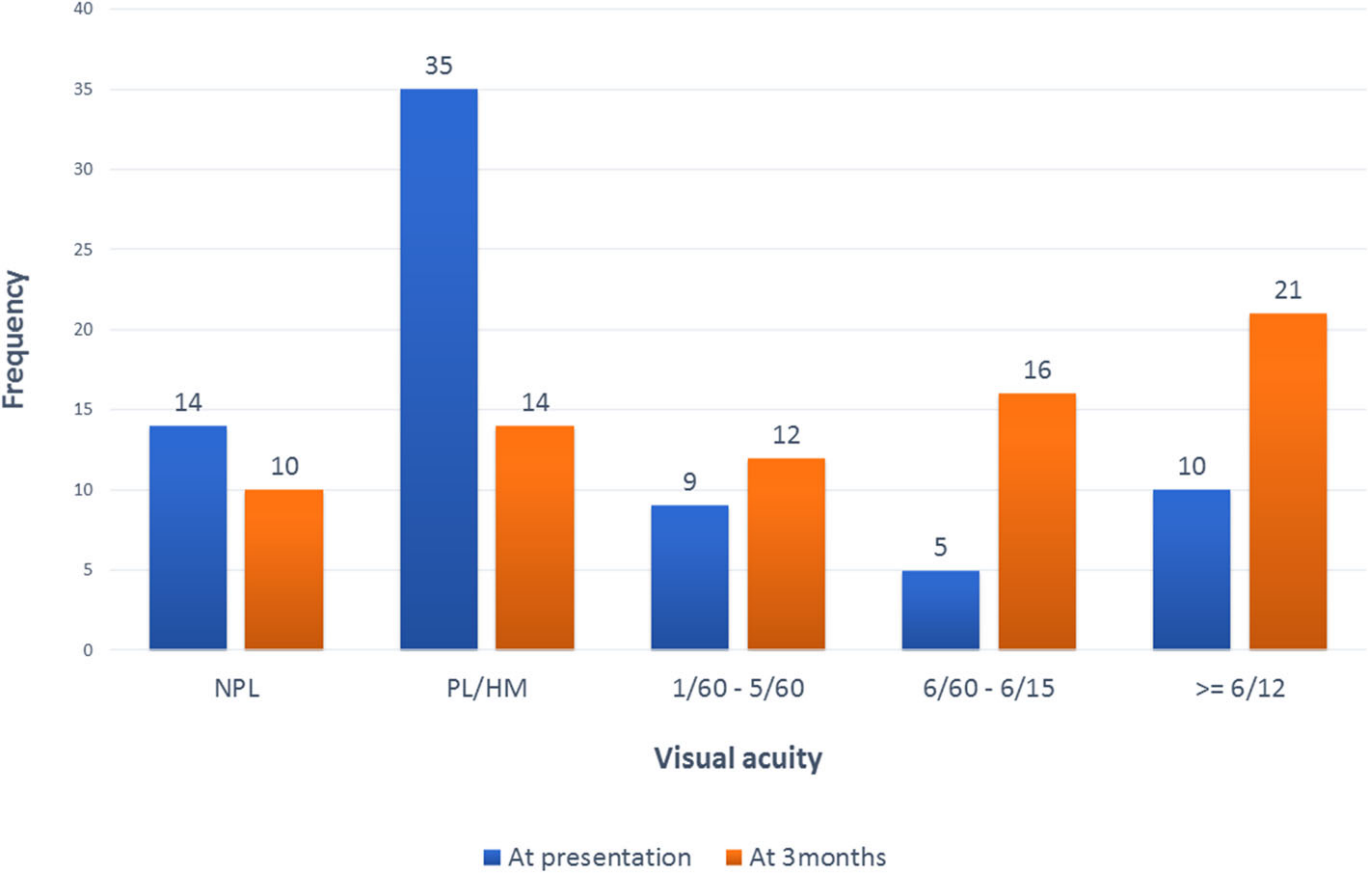
Bar diagram showing Visual acuity at presentation and at 3-month follow-up. Majority of patients presented with initial visual acuity less than 1/60, while majority of patients had visual acuity of more than 6/60 at the end of 3 months follow up. *NPL* No light perception, *PL* Perception of light, *HM* Hand movements

Visual acuity of patients at presentation and the final visual acuity attended at end of 3 months is summarized in Fig. 1. A total 56 cases (77 %) had visual acuity less than 3/60 on presentation, whereas 10 cases (15 %) had 6/12 or more. A McNemar’s Chi-squared test was applied to compare visual acuity at presentation and after three months with a statistically significant relationship [Table 3]. When a cross tab correlation analysis was done, 57.1 % of cases (8 eyes) with an initial VA of NPL had a final VA of NPL, while 90 % of cases (9 eyes) maintained a VA of 6/12 or better.

**Table 3:** Comparison of categorical distribution of visual acuity at 3-month follow-up with various risk factors

Parameters	VA at 3 months	NPL	PL/ HM	1/60–5/60	6/60 − 6/15	>= 6/12	Total	*p* value
		No. (%)	No. (%)	No. (%)	No. (%)	No. (%)	No. (%)	
** VA at presentation**	**NPL**	8(57.1)	3(21.4)	1(7.1)	2(14.3)	0(0)	14(100)	< 0.001^a^
	**PL/ HM**	2(5.7)	11(31.4)	11(31.4)	6(17.1)	5(14.3)	35(100)	
	**1/60–5/60**	0(0)	0(0)	0(0)	5(55.6)	4(44.4)	9(100)	
	**6/60–6/15**	0(0)	0(0)	0(0)	2(40)	3(60)	5(100)	
	**>= 6/12**	0(0)	0(0)	0(0)	1(10)	9(90)	10(100)	
	**Total**	10(13.7)	14(19.2)	12(16.4)	16(21.9)	21(28.8)	73(100)	
**Zones of injury**	**Zone I**	5(13.5)	3(8.1)	3(8.1)	10(27)	16(43.2)	37(100)	0.001^b^
	**Zone II**	0(0)	7(35)	5(25)	5(25)	3(15)	20(100)	
	**Zone III**	5(31.3)	4(25)	4(25)	1(6.3)	2(12.5)	16(100)	
	**Total**	10(13.7)	14(19.2)	12(16.4)	16(21.9)	21(28.8)	73(100)	
**Ruptured globes**	**Present**	4(44.4)	4(44.4)	1(11.1)	0(0)	0(0)	9(100)	0.001^b^
	**Absent**	6(9.4)	10(15.6)	11(17.2)	16(25)	21(32.8)	64(100)	
	**Total**	10(13.7)	14(19.2)	12(16.4)	16(21.9)	21(28.8)	73(100)	
**Endophthalmitis**	**Present**	6(66.7)	1(11.1)	1(11.1)	1(11.1)	0(0)	9(100)	0.001^b^
	**Absent**	4(6.3)	13(20.3)	11(17.2)	15(23.4)	21(32.8)	64(100)	
	**Total**	10(13.7)	14(19.2)	12(16.4)	16(21.9)	21(28.8)	73(100)	
**Intra vitreal injections**	**Yes**	8 (16)	12 (24)	9 (18)	13 (26)	8 (16)	50 (100)	0.013^b^
	**No**	2 (8.7)	2 (8.7)	3 (13)	3 (13)	13 (56.5)	23 (100)	
	**Total**	10 (13.7)	14 (19.2)	12 (16.4)	16 (21.9)	21 (28.8)	73 (100)	
**Time to presentation**	**<6 hours**	0 (0)	5 (16.7)	6 (20)	4 (13.3)	15 (50)	30 (100)	0.001^b^
	**6–24 hours**	3 (13)	4 (17.4)	4 (17.4)	9 (39.1)	3 (13)	23 (100)	
	**> 2 days**	7 (36.84)	25 (26.31)	2 (10.52)	2 (10.52)	3 (15.78)	19 (100)	
	**Total**	10 (13.7)	14 (19.2)	12 (16.4)	16 (21.9)	21 (28.8)	73 (100)	

*VA *Visual acuity, *NPL *No light perception, *LP *Light perception, *HM *Hand movements

^a^Mcnemar’s chi- squared test

^b^Fisher’s exact test

After calculating the final OTS score, patients were categorized into five OTS groups and final visual acuity was assessed. Most cases (31, 42 %) fell into OTS Group 3, followed by Group 2 (18, 25 %). Group 1, 4, and 5 each had 8 cases (11 %). After three months, 21 eyes (29 %) achieved a visual acuity of 6/12 or better.

### Correlation analysis

Evaluation of various risk factors for poor final visual acuity was completed [Table 3]. A Fisher’s exact test applied to compare zones of involvement with final VA was statistically significant with a p-value of 0.001. A total of 9 eyes (56.3 %) with zone III involvement had a final VA of HM or less. While VA of HM or less was found in 8 eyes (21.6 %) and 7 eyes (35 %) in patients with zones I and II involvement, respectively. The presence of a globe rupture was significantly associated with poor visual outcome when compared to non-ruptured globes (*p* = 0.001), where 8 eyes (88.8 %) with ruptured globes resulted in a VA of HM or worse compared to non-ruptured globes (16 eyes, 25 %). For patients with endophthalmitis, 7 eyes (77.8 %), had a final VA of HM or worse compared to 17 eyes (26.6 %) without endophthalmitis (*p* = 0.001). Twenty eyes (40 %) receiving intravitreal injections of antibiotics had a final VA of HM or worse, while those without injections had a VA of HM or worse in 4 eyes (17.4 %). A statistically significant relationship between time to presentation and final VA was found with a p-value of 0.001. A total of 15 patients (50 %) had a final VA of 6/12 or better when they presented within 6 hours of injury, while 3 patients (15.8 %), who presented after more than 2 days, had a final VA of 6/12 or better.

Analysis of actual final VA compared to predicted final VA based on OTS group was done using Z test for proportion. It was applied manually to each of the categories to test for the statistically significant dissimilarity. Fifteen of the 25 categories of OTS groups had a final VA with no statistically significant difference compared to the predicted outcome (*p* > 0.05) [Table 4].

**Table 4:** OTS predicted visual outcome and comparison to final visual outcome

**OTS Group**	**NPL**			**LP/HM**			**1/60 - 5/60**			**6/60 - 6/15**			**>= 6/12**		
	**OTS [%]**	**Final [%]**	*p* * value	**OTS [%]**	**Final [%]**	*p* * value	**OTS [%]**	**Final [%]**	p* value	**OTS [%]**	**Final [%]**	p* value	**OTS [%]**	**Final [%]**	p* value
**1**	74	75	***>0.05***	15	25	<0.05	7	0	<0.05	3	0	***>0.05***	1	0	***>0.05***
**2**	27	22.2	***>0.05***	26	38.9	<0.05	18	27.8	<0.05	15	11.1	***>0.05***	15	0	<0.05
**3**	2	0	***>0.05***	11	16.1	***>0.05***	15	22.6	***>0.05***	31	32.3	***>0.05***	41	29	<0.05
**4**	1	0	***>0.05***	2	0	***>0.05***	3	0	***>0.05***	22	50	<0.05	73	50	<0.05
**5**	0	0	***>0.05***	1	0	***>0.05***	1	0	***>0.05***	5	0	<0.05	94	100	<0.05

*NPL* No light perception, *LP *Light perception, *HM *Hand movements, *OTS* Ocular trauma score

^*^Z-test for proportion

## Discussion

This study describes the profile of open globe injuries presenting to a tertiary hospital in Nepal and evaluates the use of the OTS in predicting visual outcomes. The patterns of ocular trauma and demographic profile of patients found in this study closely matched to studies of ocular trauma worldwide [10, 11, 23–25]. Our study found that a large portion of the patients, about one fourth, were below the age of 10. Hence, denoting the involvement of vulnerable population. However, it must also be noted that second largest age group were between 2nd to 3rd decade of life, this may be due larger risk faced by this age group during their line of work or their frequent social activities. This correlates with the occupational distribution of patients, with more than one third being students. This study also shows the preponderance of male gender for ocular trauma. This may be explained by the fact that male population tend have more outdoor activities and have higher tendencies to face occupational hazards. Most patients were from the hilly region of Nepal, with the majority being from the urban city of Kathmandu, showing the influence of location and awareness to seek ophthalmic care.

Causative agent and mode of trauma often depend on location of a study. Similar to other studies in the rural region, the most common agent of injury was vegetative material such as sticks and branches, followed by metal; however, this is in contrast to many western studies that reported blunt objects as the most common causative agent [8, 25–27]. Accidental trauma was most common, followed by inadvertent trauma. Comparatively, studies conducted in larger urban areas of developed countries have shown assault (41 %) as the most common cause of ocular trauma [28].

Many of the variables analyzed showed a statistically significant correlation with final visual outcomes, including zone of involvement and time to presentation. The most common location of open globe injury in this population was a Zone I injury; however, more posterior injuries carried a worse prognosis similar to findings in other studies [29–31]. In general, final VA decreased as the time to presentation after trauma increased. Among the patients who presented less than 6 hours after the injury, 50 % had a final VA of 6/12 or better compared to 15.8 % of patients presenting after two days. Previous studies have also shown evidence that final visual outcome is affected by time to presentation or repair [32, 33]. However, some studies have also concluded that there may not be considerable difference in prognosis for eyes receiving early or delayed treatment [34]. In this study, it was found that initial visual acuity, extent of injury, need for intravitreal injection, development of endophthalmitis, and a ruptured globe were all predictive of a poorer final visual acuity.

When the OTS-predicted visual acuity was compared to the actual final visual acuity for each group, it was found that there was no statistically significant difference for 15 of the 25 categories, resulting in a 60 % predictive accuracy of the OTS for patients at 3 months. This is comparable to a 77 % predictive value for the OTS at 6 months in the study by Kuhn et al. [18]. The variations in Groups 1–4 captured worse outcomes than predicted, while Group 5 showed better outcomes. This may be due to the shorter follow up time that fails to reliably capture the final visual outcomes of the injured eye.

The use of the OTS in general, pediatric, weapon-related eye injuries to predict visual outcomes has been evaluated in numerous studies in North America, Europe, and Asia [4, 18, 35–38]. This is the first study in a Nepalese population and highlights both the scope of open globe injury in the Nepal and the utility of the OTS for the prediction of visual outcomes and management of patients with open globe injury.

One limitation of this study is its scope at a single hospital, which may not capture the trends of the entire population with diverse geographical settings. Further multicenter studies need to be done to better evaluate the scope of ocular injury in Nepal and utilization of the OTS in open globe injury.

## Conclusions

We found that open globe injuries presenting with poor visual acuity, delayed presentation, posterior zones of injury, need for intravitreal injections, endophthalmitis, and globe rupture are associated with poorer prognosis. OTS can be an accurate predictive tool for estimating final visual acuity even for a short follow up period of 3 months. It provides a better means for patient counseling and aids in clinical decision making; thus, OTS can be a valuable standard predictive tool for management of OGI specially in a resource limited setting of a developing country like Nepal.

## Data Availability

The datasets used and/or analyzed during the current study are available from the corresponding author on reasonable request.

## Abbreviations

OGI: Open globe injuries
OTS: Ocular trauma score
VA: Visual acuity
BPKLCOS: B.P. Koirala Lions Centre for Ophthalmic Studies
BETTS: Birmingham Eye Trauma Terminology System
RAPD: Relative afferent pupillary defect
RD: Retinal detachment
TUTH: Tribhuvan University Teaching Hospital
NPL: No perception of light
PL: Perception of light
CF: Counting fingers
